# Alterations in circadian/seasonal rhythms and vegetative functions are related to suicidality in DSM-5 PTSD

**DOI:** 10.1186/s12888-014-0352-2

**Published:** 2014-12-12

**Authors:** Liliana Dell’Osso, Gabriele Massimetti, Ciro Conversano, Carlo Antonio Bertelloni, Mauro Giovanni Carta, Valdo Ricca, Claudia Carmassi

**Affiliations:** Section of Psychiatry, Department of Clinical and Experimental Medicine, University of Pisa, Via Roma 67, Pisa, 56126 Italy; Department of Public Health, University of Cagliari, Cagliari, Italy; Department of Neuropsychiatry, University of Florence, Florence, Italy

**Keywords:** Rhythmicity, PTSD, Suicide attempts, Suicidal ideation, Vegetative functions

## Abstract

**Background:**

Alterations in rhythmicity and vegetative functions have been reported as correlates of suicidality, particularly in patients with mood disorders. No investigation has addressed their impact on patients with post-traumatic stress disorder (PTSD). Aim of the present study was to fulfill this gap.

**Methods:**

Sixty-five out- and inpatients with DSM-5 PTSD were assessed by using the Mood Spectrum-Self Report-Lifetime Version (MOODS-SR), a questionnaire for lifetime mood spectrum symptomatology including alterations in circadian/seasonal rhythms and vegetative functions. Six items of the MOODS-SR were combined and dichotomized to assess suicidal ideation and/or attempts.

**Results:**

Significant and positive associations were found between symptoms of lifetime dysregulations in rhythmicity and vegetative functions and suicidal ideation and/or attempts. All MOODS-SR sub-domains (*rhythmicity, sleep, appetite/weight, sexual function, physical symptoms*) were associated with an increased likelihood of suicidal ideation, but only changes in appetite/weight were associated with greater odd ratios of suicide attempts (OR = 2.099, 95% CI 1.148-3.841).

**Conclusions:**

Our results suggest that lifetime dysregulations in rhythmicity and vegetative functions may represent correlates of suicidality in patients with DSM-5 PTSD.

## Background

Post-traumatic stress disorder (PTSD) has been progressively investigated in the general population affected by different types of trauma and there is agreement on its tendency to a chronic course and high risk for suicide [1-9]. Data from the National Comorbidity Survey [1] showed six folds higher rates of suicide attempts in PTSD patients with respect to demographically matched control subjects.

In recent years, increasing interest has been devoted to the relationship between dysregulations in both “rhythmicity” (such as sensitivity to seasonal and circadian rhythm and weather conditions) and vegetative functions (such as sleep, appetite, sexual functions and menstrual cycle), and suicidality [10-19]. The literature on seasonal variations in the occurrence of suicide is wide and contradictory. Conversely, the impact of individual or personality characteristics, such as meteotropism, has been rarely examined, although biological (the serotonin system) and sociological (opportunities, holidays, contingencies, etc.) factors have been investigated as potential predictors of suicidal behaviour [20].

Recently, sleep alterations have been identified as potential risk factors for suicidal ideation and attempts [12,21,22]. In the National Comorbidity Survey Replication (NCS-R), the relationship between three measures of sleep patterns (difficulty in initiating sleep, maintaining sleep, early morning awakening) and suicidal ideation, plans and attempts, was explored in a sample of 5692 US adults. Sleep problems were significantly associated with suicidal ideation (OR = 2.1, CI: 1.6-2.8), plans (OR = 2.6, CI: 1.4-4.9) and attempts (OR = 2.5, CI: 1.2-5.2) [22]. Interestingly, the link between sleep problems and suicidal ideation or attempt seems to be relatively independent from the effect of psychiatric diagnoses [23-25].

Despite still scarce, some reports have explored alterations in rhythmicity and vegetative functions in PTSD. Solt et al. [26], investigating Vietnam veterans with PTSD admitted to a Veteran Hospital during a 3-year period, found admissions for PTSD to be significantly more frequent in spring and summer, suggesting the possibility that seasonal factors may influence PTSD. Sleep disturbances, including nightmares and insomnia, are core features of PTSD that likely contribute to the pathogenesis of the disorder and to poor daytime functioning [27-29]. Further, arousal regulation and memory consolidation appear to be important in determining the development of PTSD and both are functions of sleep. Somatic symptoms and sexual dysfunction may also be associated with PTSD [30-33]. Westermeyer et al. [34] explored four somatic symptoms (headaches, appetite change, dizziness, and sleep problems) in 622 Somali refugees finding significantly higher scores in survivors with PTSD, particularly those who experienced more types of trauma or higher symptoms scores.

In the framework of the Spectrum Project (a USA-Italy collaboration), a questionnaire was developed and validated, that explores a full spectrum of lifetime mood spectrum phenomenology (Mood Spectrum-Self Report, MOODS-SR) [35] including dysregulations in rhythmicity and vegetative functions. The MOODS-SR rhythmicity and vegetative functions domain proved to be associated with suicidality in patients with schizophrenia and unipolar, bipolar, borderline personality and panic disorder [36]. Moreover, in a previous study some of us demonstrated that, amongst vegetative functions, dysregulations in sexual functioning and behavior are significantly associated with suicidality in patients with mood disorders [37].

The aim of the present study was to investigate dysregulations in rhythmicity and vegetative function, as assessed by the MOODS-SR, in patients with PTSD, and their impact on suicidality.

## Methods

### Design

A consecutive sample of 65 out- and inpatients of both sexes (33 men and 32 women, mean age ± SD: 45 ± 14.8 years), with a DSM-IV-TR diagnosis of chronic PTSD, was recruited at the Section of Psychiatry of the Department of Clinical and Experimental Medicine, University of Pisa, Italy. Methods and sample characteristics were described in details in a previous study [38]. The majority of patients were married or living with a partner (N = 37, 56.9%), and had more than 8 years of education (N = 38, 58.4%). Thirty-eight patients (58.4%) were employed full-time or part-time and the remaining were unemployed or retired (see Table 1).

**Table 1:** **Demographic characteristics of PTSD patients (N = 65)**

	***N (%)***
***Gender***	
*Women*	32 (49.2)
*Men*	33 (50.8)
***Marital status***	
*Single*	15 (23.1)
*Married/living with partner*	37 (56.9)
*Widows-ers*	4 (6.2)
*Separated/divorced*	9 (13.8)
***Educational level achieved***	
*Primary school (5 years)*	9 (13.8)
*Secondary school (8 years)*	18 (27.7)
*Professional school (12 years)*	3 (4.6)
*High school diploma (13 years)*	28 (43.1)
*University degree (> = 16 years)*	7 (10.8)
***Occupation***	
*Employed*	38 (58.4)
*Unemployed*	6 (9.2)
*Retired*	13 (20.0)
*Other*	8 (12.4)

All subjects were reassessed for DSM diagnosis according to DSM-5 criteria by clinical researchers expert in the field.

The Ethics Committee of the Azienda Ospedaliero-Universitaria of Pisa approved all recruitment and assessment procedures. Participants provided written informed consent, after receiving a complete description of the study and having the opportunity to ask questions.

### Diagnostic assessment

Assessments included the SCID-IV-TR and the MOODS-SR lifetime version.

The MOODS-SR, a questionnaire exploring mood spectrum symptomatology [35], includes 161 items coded as present/absent, for one or more periods of at least 3 to 5 days across the lifespan. Items are organized into 3 manic and 3 depressive domains, exploring “mood”, “energy” and “cognition”, besides a “rhythmicity and vegetative functions” domain. The manic and depressive domains focus on the presence of specific manic and depressive features respectively, including either isolated or clustered typical and atypical symptoms, traits and lifestyles that may characterize the temperamental affective dysregulations that make both fully syndromal and subthreshold mood disturbances. The rhythmicity and vegetative functions domain includes 30 items organized into 5 subdomains that explore seasonal or circadian variations in mood and energy (“rhythmicity”), changes in weight/appetite, sexual activity, sleep and physical symptoms (“vegetative functions”). All MOODS-SR domains demonstrated a good internal consistency with Kunder-Richardson coefficients values always exceeding 0.79 [35].

The *rhythmicity* subdomain consists of 6 items investigating alterations in mood, energy and physical well-being according to the weather, the season, and the phase of menstrual cycle. The vegetative functions subdomains are: *sleep* (12 items), *weight and appetite* (4 items), *sexual functions* (5 items), *physical symptoms* (5 items, including headaches, dry mouth, constipation and stomach or bowel problems and sensitivity to heat, cold or pain). For the present study we excluded from the analyses two items of the sleep subdomain that explore alterations over the course of the menstrual cycle and apply only to females. The instrument can be downloaded from the web site www.spectrum-project.org. Suicidality is assessed using 6 items of the MOODS-SR that explore whether the subject had *ever experienced periods of 3 to 5 days or more when he or she*: *thought that life is not worth living (N=102)*; *wished he/she would not wake up in the morning, or that he/she would die in an accident or from something like a heart attack or a stroke (N=103)*; *wanted to die or hurt him/herself (N=104)*; *wanted to die and had a specific plan to hurt or kill him/herself (N=105)*; *actually committed a suicide attempt (N=106)*; *committed a suicide attempt that required medical attention (N=107)*. For the purpose of the present study, suicidality was rated counting the positive answers to these questions.

### Statistical analyses

Chi squared tests were utilized to compare the frequencies of endorsement of the rhythmicity and vegetative functions items of PTSD patients with at least one positive suicidal item on the MOODS-SR compared to those without suicidality. Student’s t-tests were utilized to compare the same two groups for what concern rhythmicity and vegetative functions domain and subdomains scores. The relationship between suicidality and the rhythmicity and vegetative functions domain total score was investigated with an univariate linear regression analysis. The relationships between suicidality and the rhythmicity and vegetative function sub-domains scores were investigated by using a multiple linear stepwise regression model.

Statistical analyses were carried out using the Statistical Package for Social Science [39], version 20.0.

## Results

At the index assessment, 20 (30.8%) PTSD patients, out of the total of 65, also met the DSM-5 criteria for major depression. On average, participants endorsed 10.2 items (SD = 5.5) on the rhythmicity and vegetative functions domain.

The frequency of endorsement of suicidality items was: 46.2% (*thought that life is not worth living*); 27.7% (*wished he/she would not to wake up in the morning, or that he/she would die in an accident or from something like a heart attack or a stroke*); 35.4% (*wanted to die or hurt him/herself*); 10.8% (*wanted to die, had a specific plan to hurt or kill him/herself*); 12.3% (*actually committed a suicide attempt*); and 7.7% (*the attempt required medical attention*)*.*

The frequency of endorsement of rhythmicity and vegetative function items is reported in Table 2. The most frequently endorsed items were hypersensitivity to rhythm disruptions and sleep problems. In the same table the frequency of endorsement of the same items was compared between PTSD patients with at least one suicidal item with respect to those with no suicidality. Results showed significantly higher percentages in all the appetite/weight subdomain items and in about half of the sleep rhythmicity and physical symptoms sub-domain items. The comparisons between PTSD patients with at least one suicidal item with respect to those with no suicidality in the rhythmicity and vegetative functions domain total and subdomains scores are reported in Table 3. Statistically significant higher scores emerged in PTSD patients with at least one suicidal item in all scores.

**Table 2:** **MOODS-SR rhythmicity and vegetative functions items endorsement in the total sample (n = 65) and in PTSD patients without MOODS-SR suicidal items (n = 29) with respect to those with at least 1 suicidal item (n = 36)**

**MOODS-SR rhythmicity item**		***Total sample N(%)***	***No MOODS-SR suicidal items N(%)***	***≥ 1 MOODS-SR suicidal item N(%)***	***p***
132	Difficulty working in the early morning	17 (26.2)	5 (17.2)	12 (33.3)	.237
133	Difficulty working in the evening or night	24 (36.9)	6 (20.7)	18 (50.0)	.030
134	Irritable if daily routine is disrupted	20 (30.8)	4 (13.8)	16 (44.4)	.017
135	Mood, energy, interest and efficiency improved if you were in a regular routine	36 (55.4)	13 (44.8)	23 (63.9)	.199
136	More energetic with less sleep	14 (21.5)	1 (3.4)	13 (36.1)	.004
137	Mood, energy and physical well-being changing with the weather, season or when travelling across time zones	42 (64.6)	16 (55.2)	26 (72.2)	.243
Sleep					
138	Sleepy all the time	26 (40.0)	5 (17.2)	21 (58.3)	.002
139	Repeated difficulty falling asleep	42 (64.6)	14 (48.3)	28 (77.8)	.027
140	Repeatedly waking up in the middle of the night	42 (64.6)	13 (44.8)	29 (80.6)	.006
141	Repeatedly waking up early	38 (58.5)	12 (41.4)	26 (72.2)	.024
142	Needing much more sleep than usual	31 (47.7)	7 (24.1)	24 (66.7)	.002
143	You went for days without sleeping or with much less sleep than usual but didn’t feel tired?	22 (33.8)	8 (27.6)	14 (38.9)	.488
144	Difficulty sleeping before or after stimulating activities	28 (43.1)	11 (37.9)	17 (47.2)	.617
145	Increase in quality/need for sleep in a particular season	25 (38.5)	6 (20.7)	19 (52.85)	.017
146	Increase in quality/need for sleep when traveling across > = 4 time zones	8 (12.3)	4 (13.8)	4 (11.1)	1.00
147	Decrease in quality/need for sleep when traveling across > = 4 time zones	8 (12.3)	3 (10.3)	5 (13.9)	.723
Appetite/Weight					
150	No food appeals or tastes good	23 (35.4)	6 (20.7)	17 (47.2)	.050
151	Constantly craving for sweets or carbohydrates	20 (30.8)	3 (10.3)	17 (47.2)	.003
152	Appetite/weight increased	16 (24.6)	2 (6.9)	14 (38.9)	.007
153	Appetite/weight decreased	21 (32.3)	3 (10.3)	18 (50.0)	.002
Sexual function					
154	Less sexually active than usual	27 (41.5)	7 (24.1)	20 (55.6)	.021
155	Difficulty becoming sexually aroused	25 (38.5)	7 (24.1)	18 (50.0)	.061
156	Difficulty achieving orgasm	13 (20.0)	5 (17.2)	8 (22.2)	.852
157	More interested in sex	11 (16.9)	2 (6.9)	9 (25.0)	.109
158	Frequently changing sexual partners	8 (12.3)	1 (3.4)	7 (19.4)	.066
Physical symptoms					
159A	Frequent headaches	25 (38.5)	5 (17.2)	20 (55.6)	.004
159B	Dry mouth	27 (41.5)	5 (17.2)	22 (61.1)	.001
159C	Constipation	20 (30.8)	7 (24.1)	13 (36.1)	.442
159D	Nausea or other GI problems	25 (38.5)	7 (24.1)	18 (50.0)	.061
160	More/less sensitive than usual to heat, cold or pain	29 (44.6)	6 (20.7)	23 (63.9)	.001

**Table 3:** **MOODS-SR rhythmicity and vegetative functions domain and subdomains scores in PTSD patients without MOODS-SR suicidal items (n = 29) compared with those with at least 1 suicidal item (n = 36)**

**MOODS-SR rhythmicity and vegetative functions domain**	***No MOODS-SR suicidal items (mean ± SD)***	***≥ 1 MOODS-SR suicidal item (mean ± SD)***	***p***
**Total score**	6.34 ± 4.48	13.33 ± 4.20	< .001
**Subdomains**			
*Rhytmicity*	1.55 ± 1.33	3.00 ± 1.59	< .001
*Sleep*	2.86 ± 2.26	5.19 ± 2.01	< .001
*Appetite/weight*	0.48 ± 0.87	1.83 ± 1.16	< .001
*Physical symptoms*	1.04 ± 1.70	2.67 ± 1.88	.001
*Sexual function*	0.76 ± 1.12	1.72 ± 1.23	.002

**Figure 1:**
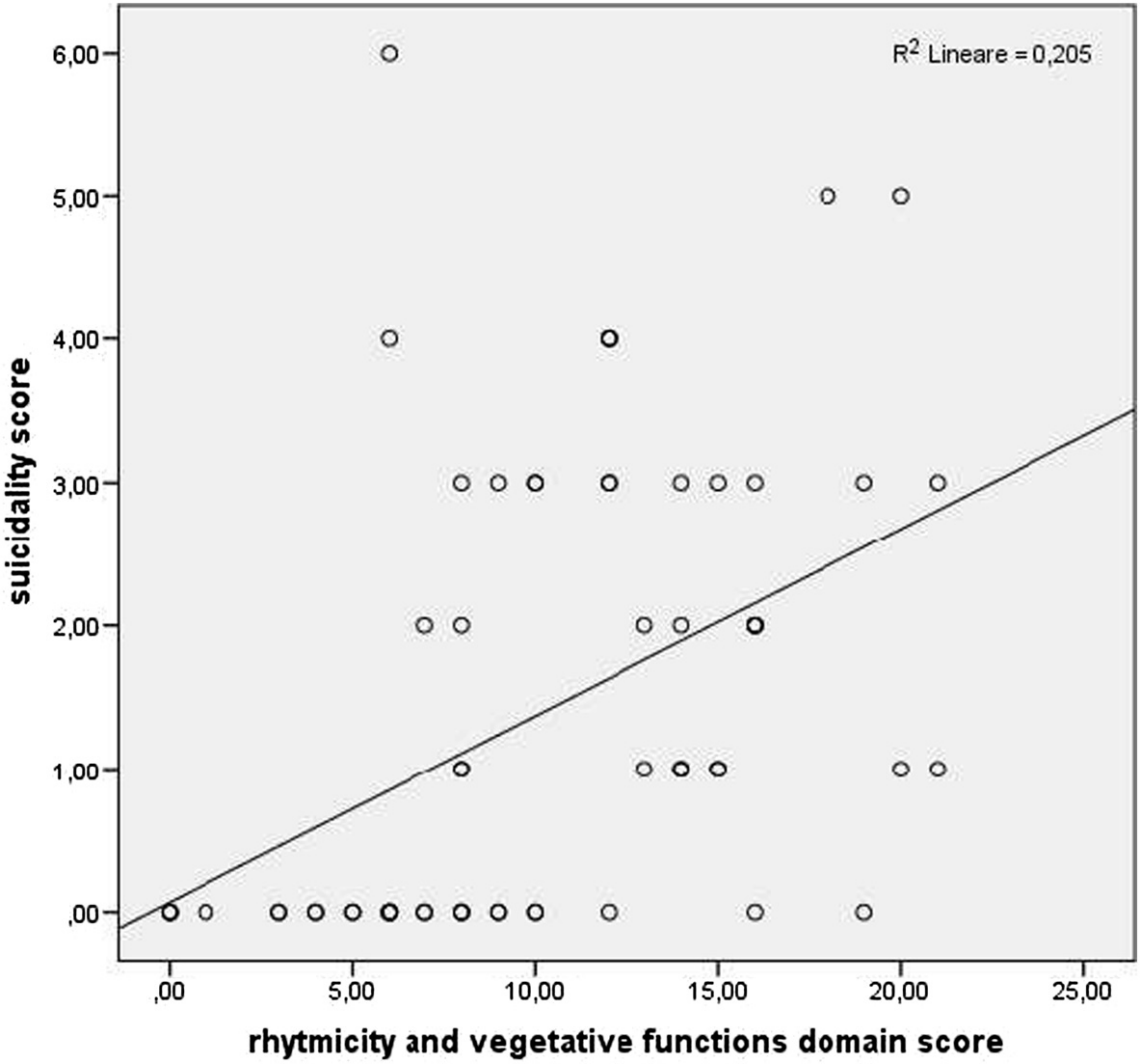
**Association between MOODS-SR rhythmicity and vegetative functions domain score and suicidality.**

A linear regression analysis showed a statistically significant association between the rhythmicity and vegetative functions domain scores and suicidality [b = 0.13 (SE = 0.03, β = 0.45, t = 4.02, p < 0.001)] (Figure 1).

An exploratory multiple linear regression analysis stepwise model was adopted in order to detect the most affecting subdomains. A significant association emerged between suicidality and first the appetite/weight subdomain and secondly the rhythmicity subdomain (see Table 4).

**Table 4:** **Stepwise multiple linear regression analysis in 65 PTSD patients: MOODS-SR rhythmicity subdomains scores predicting suicidality**

**Step**	**MOOD-SR rhythmicity subdomain scores**	***b (SE)***	***ß***	***R*** ^***2***^	***t***	***p***
*1*	Constant	0.605 (0.246)		.249	2.46	.016
	Appetite	0.646 (0.141)	.499		4.57	< .001
*2*	Constant	0.056 (0.316)		.323	0.18	.0861
	Appetite	0.571 (0.138)	.441		4.13	<.001
	Rhythmicity	0.272 (0.105)	.278		2.61	.011

As the study group included 20 patients with a current episode of major depression and another 14 who had been suffering from past depressive, we hypothesized that the association between rhythmicity and vegetative functions symptoms and suicidality might be confounded by depression. Therefore, we preformed the linear regression analysis in the subsample of patients without current or past major depression. The rhythmicity and vegetative functions total score was still significantly associated with suicidality [b = 0.09, (SE = 0.05, β = 0.35, t = 2.04, p = .05)].

## Discussion

The results of the present study indicate high rates of impaired rhythmicity and vegetative functions, reported across the lifespan, in patients with PTSD. In particular, sleep problems and hypersensitivity to disruptions in circadian and/or seasonal rhythms were the most frequent, with more than half of the patients reporting such symptoms.

Sleep disturbances represent a core feature of PTSD. Nightmares and insomnia are diagnostic symptoms of PTSD (DSM-5) [40-42] and other sleep disturbances, such as sleep avoidance, sleep terrors, nocturnal anxiety attacks, simple and complex motor behaviors and vocalizations, acting out dreams, sleep apnea and periodic leg movement disorders, are also frequently reported by and observed in PTSD patients [43-45]. Despite a major limitation of the present study is the use of a lifetime instrument such as the MOODS-SR that does not allow discriminating whether sleep disturbances occurred before or after the trauma exposure, we can speculate that patients at higher suicidal risk are more prone to report sleep problems across the lifespan. In this regards, subjective and objective sleep disturbances occurring early after trauma exposure have been reported to be associated with an increased risk for meeting criteria for PTSD at subsequent assessments conducted one year later [46,47]. Previous data have shown that sleep disturbances exacerbate daytime symptoms and contribute to poor outcomes in PTSD, such as increased severity of depression, suicidality, and general physical distress, poorer quality of life and functioning, poorer perceived physical health and increased alcohol or drug use [45,48-51]. Our results may be considered consistent with these previous findings giving further evidence that lifetime sleep disturbances are predictive of increased suicidal ideations in PTSD.

While little is known on alterations in rhythmicity in patients with PTSD, several authors highlighted the association between alterations in circadian and/or seasonal rhythms and suicide. Increased sensitivity to changes in weather and season has been related to higher risk for suicide in the general population [11,17,20], but to the best of our knowledge, this is the first study focusing on the presence and impact of such symptoms in PTSD patients. Results from previous studies based on the MOODS-SR assessment, indicate a positive association between the number of lifetime rhythmicity and vegetative functions dysregulations and an increased risk for suicidal ideation/plans in patients with unipolar depression and borderline personality disorder. Our data are in line with studies suggesting that higher levels of suicidality are related not so much to the weather or the season “per se” but rather to the peculiar sensitivity to changes of weather and season [36].

It is noteworthy that our correlations were confirmed even after controlling for depressive comorbidity suggesting the role of lifetime disturbances in rhythmicity as a possible correlate of suicidality in PTSD patients independently from mood disorder comorbidity. There is evidence that mood disorders, and particularly bipolar disorders, are sensitive to the environmental influence in general and to the seasonal effect in specific, and frequently report alterations in vegetative functions such as sleep, appetite, and sexual functions [52-56]. Thus, our data suggest a lifetime subthreshold bipolar comorbidity to be related to a higher suicidality in PTSD, similarly to what happens for comorbidity with full blown bipolar disorder [57-59]. Consistently, in a previous report on PTSD patients without bipolar disorder comorbidity, we found statistically significant and positive associations between manic/hypomanic and depressive symptoms and the likelihood of suicidal ideation or attempts suggesting an impact of even subthreshold forms of bipolar disorder [60]. Similarly, other authors suggested theoretical models highlighting clinical symptoms that could lead to suicidality (i.e., through feelings of hopelessness, defeat and entrapment) [61,62].

Different data would indicate a relationship between a specific and particularly severe trauma, such as childhood sexual abuse (CSA), and abnormal eating attitudes and behaviors [63,64]. The same association has been reported with other forms of victimization, trauma and neglect, including, but being not limited to, sexual assault [65-67]. In sum, these studies suggest that any experience that can produce PTSD may increase the probability of developing threshold or subthreshold eating disorders. On this line, some authors hypothesized that eating disorders could represent, for a traumatized patient, an attempt to regulate the overwhelming affective states, and that bingeing and purging may be used in order to control PTSD symptomathology [68,69]. Our findings, besides showing the high occurrence of changes in weight and appetite in PTSD patients, suggest that these symptoms may confer a greater risk for suicidal ideation and suicide attempts across the lifespan, irrespective of previous or current major depression, and that they should be carefully considered during the routine clinical assessment. It is noteworthy that the link between diagnosable eating disorders and suicidal behaviors has been recognized for some time [65], whereas recent evidence from studies in adolescents suggests a correlation between suicidal behavior and disordered eating behaviors that fall short of the threshold for an eating disorder diagnosis [66,67]. Our data extend this evidence to adults, and taken together, stress the increased risk for suicide in those adult PTSD patients displaying eating changes in weight and appetite, which should be therefore. We could also argue that the links between alterations in appetite and/or weight and suicide attempts point towards some role for disturbed serotonergic neurotransmission, since changes in appetite and craving for sweets and carbohydrates can be influenced by serotonin. Moreover, these symptoms frequently occur in patients with bipolar disorder, and this might be a further indicator of soft bipolarity in these patients [70-72].

Several limitations of the study are important to note. First of all, as already mentioned, this was a cross-sectional study in which the use of a lifetime assessment does not allow establishing whether the alterations in rhythmicity and vegetative function preceded or co-occurred with suicidal ideation or attempts. No data, in fact, was collected in the study that could provide information as to whether a temporal association exists between these two variables. Second, rhythmicity and vegetative functions symptoms were assessed by means of a self-report and this may be less reliable than objective observations. Matousek et al. [73] reported poor agreement between the subjective and objective assessment of disturbed night sleep and alertness in the daytime in a sample of patients with mild depression, suggesting a significant influence of anxiety and depression on these reports. Similarly, the presence of suicidal ideation or attempts was not assessed through the rating of the clinician, so that a self-report of suicidality may be considered less accurate. In any case, literature data comparing self-reports and clinical assessment of suicidal symptoms indicate that self-rating of suicidality contains considerable predictive value and should be the primary data source [38]. A further limitation relates to the measure of suicidal ideation used in study. This measure was a composite score of a range of behaviors including wishes of death, suicidal ideation and planning. Particularly, wishes of deaths might represent different phenomena from suicidal thoughts and plans and, although the continuum view of suicidality has received support by recent studies [74], this may represent an important methodological limitation. Third, the sample size was small, although with a similar number of men and women. Fourth, because the MOODS-SR is a lifetime assessment we could not determine whether an ongoing drug treatment might have influenced the alterations in weight and/or appetite reported. Fifth, the lack of information on the characteristics of the index trauma (e.g. severity, degree of exposure, length of exposure) and of PTSD in terms of chronicity of the illness, may affect the results of the present study as they could not be generalizable to all categories of patients diagnosed with PTSD.

## Conclusions

In conclusion, despite taking into account the limitations mentioned above, our results suggest that impaired rhythmicity and vegetative functions correlate with lifetime suicidality in PTSD patients. Therefore, our data highlight the need to explore these functions in PTSD patients across the lifespan, in order to identify as soon as possible individuals at higher suicidal risk.
